# Microparticles from patients with systemic lupus erythematosus induce production of reactive oxygen species and degranulation of polymorphonuclear leukocytes

**DOI:** 10.1186/s13075-017-1437-3

**Published:** 2017-10-17

**Authors:** Line Kjær Winberg, Søren Jacobsen, Claus H. Nielsen

**Affiliations:** 1 Institute for Inflammation Research, Center for Rheumatology and Spine Diseases, Copenhagen University Hospital, Rigshospitalet, Section 7521, Copenhagen, Denmark; 2 Copenhagen Lupus and Vasculitis Clinic, Center for Rheumatology and Spine Diseases, Copenhagen University Hospital, Rigshospitalet, Section 4242, Blegdamsvej 9, DK-2100 Copenhagen, Denmark

## Abstract

**Background:**

The interaction of circulating microparticles (MPs) with immune cells in systemic lupus erythematosus (SLE) is sparsely investigated. We examined the ability of MPs from SLE patients to induce production of reactive oxygen species (ROS) and degranulation of polymorphonuclear leukocytes (PMNs).

**Methods:**

Plasma MPs, leukocytes and sera isolated from 20 SLE patients and 10 healthy controls were mixed in different combinations, with or without lipopolysaccharide (LPS), and incubated for 30 min. Dihydrorhodamine 123 was used to measure ROS production by flow cytometry. The ability of immunoglobulin G (IgG) isolated from five SLE patients to increase MP-induced production of ROS by PMNs was tested. Cell supernatants were analysed for content of primary, secondary and tertiary granule components by Luminex assays.

**Results:**

MPs from SLE patients promoted ROS production by PMNs, and enhanced LPS-induced ROS production and release of primary granules by PMNs, when added to samples of autologous leukocytes and serum. In a similar autologous setting, MPs from healthy controls enhanced LPS-induced ROS production by PMNs. When leukocytes from a healthy control were stimulated with autologous MPs in the presence of various sera, SLE patient serum promoted ROS production and release of primary and secondary granules by PMNs. A role for antibodies in this respect was indicated by the observation that supplementation of normal serum with IgG from SLE patients promoted MP-induced ROS production by healthy PMNs. Moreover, when various MPs were incubated with leukocytes and serum from a healthy control, patient-derived MPs induced more ROS production by PMNs than did healthy control-derived MPs.

**Conclusions:**

SLE patients display increased ROS production and degranulation by PMNs in response to MPs, which partly depends on serum components, including antibodies, MP properties and hyper-responsiveness of the PMNs per se.

**Electronic supplementary material:**

The online version of this article (doi:10.1186/s13075-017-1437-3) contains supplementary material, which is available to authorized users.

## Background

Systemic lupus erythematosus (SLE) is a systemic autoimmune disease of unknown aetiology, characterized by the presence of a multitude of circulating autoantibodies against components of cellular origin which includes a variety of anti-nuclear antibodies. There is no consensus on how nuclear antigens are presented to the immune system, but circulating microparticles (MPs) carrying cellular constituents are among the main candidates [1]. MPs are small extracellular vesicles in the range of 0.1–1 μm, shed from apoptotic or activated cells by blebbing of the cell membrane. They have known effects on thrombosis, vasculature and inflammation [2], and alterations in MP concentration and composition have been linked to autoimmunity, especially SLE [3, 4]. Detection of autoantibodies binding to nuclear material on the surface of MPs in SLE and findings suggestive of MP deposition on the glomerular basement membrane in patients with lupus nephritis indicate that MPs play a central pathogenic role in SLE [5, 6]. Whereas many studies have focused on surface properties of MPs, including autoantigenic properties, the interactions between MPs and immune cells, and the subsequent consequences in terms of cellular functions, are sparsely described.

Evidence of involvement of polymorphonuclear leukocytes (PMNs) in the pathogenesis of SLE has become increasingly established during the past decade: PMNs from SLE patients show enhanced apoptosis and production of neutrophil extracellular traps (NETosis) [7] and decreased production of reactive oxygen species (ROS) after stimulation with phorbol-12-myristate-13-acetate (PMA) [8], as well as with complement-opsonized immune complexes (ICs) [9]. However, this may be explained by a generalized hyper-reactivity of SLE PMNs [7, 10, 11] and death of the most reactive PMNs [8]. Increased oxidative stress has been linked with SLE and may contribute to immune dysregulation including increased occurrence of post-translational modifications leading to modified self-antigens and autoantibody production [12]. Further, antioxidant treatment has been shown to reduce both oxidative stress and symptom severity in SLE [13, 14].

The main receptors involved in PMN ROS production elicited by complement-opsonized ICs are complement receptor 3 (CR3, CD11b/CD18) in concert with IgG-Fc receptor (FcγR) II [15, 16]. Stimulation with complement-opsonized ICs also leads to complement-receptor-independent release of elastase from azurophilic (primary) granules and CR3-mediated release of lactoferrin from specific (secondary) granules [15]. Other markers of primary and secondary granules are myeloperoxidase (MPO) and neutrophil gelatinase-associated lipocalin (NGAL), respectively [17]. Whereas FcγRII is involved in both ROS generation and degranulation, engagement of FcγRIII seemingly results only in degranulation [18].

We recently showed that incubation of MPs from SLE patients with autologous leukocytes leads to deposition of MPs on PMNs, which is greater than the corresponding deposition of MPs from healthy controls on autologous PMNs [19]. The implications of this deposition for PMN function are largely unknown, and existing studies have primarily investigated effects of in-vitro-generated MPs [20, 21]. For example, one study showed increased phagocytic activity and CR3 expression by PMNs after incubation with platelet-derived, in-vitro-generated MPs [20]. The immune stimulatory potential of plasma MPs has been investigated by Dieker et al. [22], who demonstrated that MPs from SLE patients primed neutrophils for LPS-induced NETosis. Given MPs’ similarity with ICs in terms of carriage of IgG and complement components [19, 23], we speculated that they may be capable of eliciting ROS production and degranulation of PMNs. MPs might thereby contribute to the increased oxidative stress in SLE patients. In this study, we investigated the ability of MPs to induce ROS production and degranulation in PMNs, and we determined whether these responses differ between SLE patients and healthy controls.

## Methods

### SLE patients and healthy controls

SLE patients were included from our inpatient and outpatient rheumatology clinic and fulfilled internationally accepted classification criteria [24, 25]. Disease activity was described using the Systemic Lupus Erythematosus Disease Activity Index (SLEDAI)-2 K 30 days [26] and other available clinical information. The healthy controls included were anonymous blood donors at the Blood Bank of Copenhagen University Hospital, Rigshospitalet. The study was approved by the regional scientific ethics committee (protocol no. H-1-2013-046). For experiments including only SLE and healthy control sera or MPs as variables, frozen samples from this study and an earlier study [19] were used. Patient characteristics are presented in Table 1.

**Table 1:** Characteristics of included SLE patients and HCs

	Fresh cells, serum and MPs	Frozen serum and MPs
SLE patients	20	28
Age, median (range)	45 (22–64)	42 (22–69)
Sex, number (%) of females	17 (85)	24 (86)
Disease manifestations at study inclusion, number (%) of patients		
Renal disease	2 (10)	8 (29)
Vasculitis	2 (10)	3 (11)
Arthritis	2 (10)	3 (11)
Interstitial lung disease	1 (5)	0 (0)
Haemolysis	1 (5)	1 (4)
Alopecia	3 (15)	1 (4)
Rash	2 (10)	3 (11)
Mucosal ulcers	3 (15)	3 (11)
Leucopenia	1 (5)	1 (4)
Serositis	0 (0)	1 (4)
Thrombocytopenia	0 (0)	1 (4)
SLEDAI, median (range)	4 (0–12)	5 (0–18)
HCs	10	11
Age, median (range)	33.5 (25–63)	34 (24–54)
Sex, number (%) of females	6 (60)	9 (82)

*HC* healthy control, *SLE* systemic lupus erythematosus, *SLEDAI* Systemic Lupus Erythematosus Disease Activity Index

### Purification of MPs

MPs were purified as described previously [19]. In brief, 6 ml of blood was collected in a K_2_EDTA tube (Vacuette; Greiner Bio-one GmbH, Kremsmünster, Austria) by venous puncture. After transfer of 600 μl of the blood to other tubes for the purification of leukocytes, as described later, the blood was centrifuged at 1800 × *g* for 10 min at 37 °C and supernatants were transferred to a new tube and centrifuged at 3000 × *g* for 10 min at 37 °C for removal of platelets. The platelet-poor plasma was then filtered through a 1.2-μm syringe filter (Minisart; Sartorius, Göttingen, Germany) and divided into aliquots of 460 μl. Each aliquot was added 10% RPMI-1640 (Gibco, Thermo Fisher Scientific, Waltham, MA, USA) and ultracentrifuged twice at 19,000 × *g* for 30 min at 21 °C; the first step was followed by washing in RPMI, and the last step was followed by resuspension in RPMI at a total volume of 100 μl.

### Purification of immunoglobulin from SLE patients

A pool of sera from five SLE patients was transferred to an Illustra Column PD-10 (GE Healthcare, Chicago, IL, USA) containing 4 ml rProtein A Sepharose Fast Flow (Amersham Biosciences, GE Healthcare), and after standing 10 min at 4 °C the column was rinsed with 4 × 10 ml of Dulbecco’s phosphate buffered saline (PBS; Biological Industries, Cromwell, CT, USA). After adding 5 × 1 ml of glycine buffer (0.2 M, pH 2.4), the sample was transferred to a Vivaspin 15R10 kD (Sartorius) and centrifuged at 3000 × *g* for 10 min at 22 °C. After washing with 5 ml PBS, the centrifugation was repeated and the remaining 500 μl sample was resuspended in a total of 2 ml PBS. Lastly, the sample was transferred to a Float-A-Lyzer G2 Dialysis Device 50 kD (Spectrum Laboratories Inc., Rancho Dominguez, CA, USA) and dialysed over night against 500 ml PBS that was renewed once. The protein concentration of the dialysate was measured to be 10.7 mg/ml using a Spectro Star Nano (BMG Labtech, Ortenberg, Germany) with an LVis Plate (BMG Labtech).

### Stimulation of PMNs

Aliquots of 200 μl K_2_EDTA blood were stored at 4 °C for approximately 1 hour, until the last step of the MP purification. Afterwards, the red blood cells were removed by 15 min of lysis in a 1:10 dilution of BD Pharm Lyse Lysing Buffer (BD Biosciences, San Jose, CA, USA), according to the manufacturer’s instructions. After lysis, the solution was centrifuged twice at 300 × *g* for 5 min at room temperature; the first step was followed by washing in Dulbecco’s Phosphate Buffered Saline (Thermo Fisher Scientific), and the final volume was adjusted to 200 μl. Aliquots of 36.6 μl of the cell solution were incubated for 30 min at 37 °C in RPMI-1640 containing 25% (v/v) serum at a total volume of 200 μl with no stimulation, 10 μg/ml E. Coli LPS (Sigma, Merck, Kenilworth, NJ, USA), 20 μl of purified MP solution, 10 μg/ml LPS plus 20 μl of purified MP solution or 60 nM PMA (Sigma). All samples contained 0.66 mM dihydrorhodamine 123 (DHR) (Molecular Probes, Thermo Fisher Scientific), which is oxidized intracellularly by H_2_O_2_ to fluorescent rhodamine 123. An additional aliquot was incubated without DHR to measure background fluorescence. After incubation, the samples were washed in cold PBS and centrifuged at 740 × *g* for 10 min at 4 °C. Supernatants were removed for analysis of granule content (see later) and cells were incubated with anti-CD15 APC (BD Biosciences) for 30 min at 4 °C. The LIVE/DEAD Fixable Near-IR Dead Cell Stain (Molecular Probes) was used to discriminate between live and dead PMNs. After a similar wash, cells were analysed by flow cytometry using a BD FACSCanto II (BD Biosciences). Additional file 1 shows the gating strategy.

In another series of experiments, leukocytes from SLE patients and healthy controls were stimulated with the previously described stimuli in the presence of either autologous serum or normal human serum (NHS) from of a pool of sera from blood group AB-positive donors (Sigma Aldrich, Merck). In a third series, leukocytes from a single healthy donor were stimulated in the presence of various sera from SLE patients or healthy controls. In a fourth series of experiments, leukocytes from healthy controls were stimulated in RPMI-1640 containing autologous serum supplemented with IgG purified from sera of five SLE patients, as already mentioned, or with normal IgG for intravenous use (IVIg; CSL Behring, King of Prussia, PA, USA). In a fifth series of experiments, leukocytes from a healthy control, suspended in RPMI containing autologous serum, were stimulated with MPs from various SLE patients or healthy controls. Table 2 presents an overview of the different experimental conditions applied.

**Table 2:** Overview of experiments

Experiment/Figure	PMNs	Serum	MPs	LPS	IgG
1	Autologous SLE and HC	Autologous SLE and HC	Autologous SLE and HC	+/–	–
2	Autologous SLE and HC	SLE/normal human serum	Autologous SLE and HC	+/–	–
3	HC	SLE/HC	HC	+	–
4	HC	HC	HC	+/–	SLE/normal
5	HC	HC	SLE/HC	+	–

*HC* healthy control, *IgG* immunoglobulin G, *LPS* lipopolysaccharide, *MP* microparticle, *PMN* polymorphonuclear leukocyte, *SLE* systemic lupus erythematosus

### Measurement of degranulation

Supernatants were kept frozen at –80 °C until analysis of granule content using R&D Magnetic Luminex Screening Assays (R&D Systems, Minneapolis, MN, USA) on a Luminex Bio-Plex® 200 system (Bio-Rad Laboratories, Hercules, CA, USA). The content of MPO from primary granules was analysed with the aid of a one-plex MPO, and the content of NGAL and gelatinase (MMP-9) from secondary and tertiary granules, respectively, was analysed using a two-plex MMP-9 and Lipocalin-2/NGAL. Analysis of the fluorescence data was done using Bio-Plex Manager 6.0 Software (Bio-Rad Laboratories). The experiments were carried out according to the manufacturer’s instructions.

### Statistical analysis

Because the study data were not normally distributed, non-parametric statistical methods were applied. The effects of different stimuli were investigated using the Wilcoxon matched-pairs signed rank test. Comparisons between groups were done using the Mann–Whitney *U* test, and correlations were determined using Spearman’s correlation coefficient (*r*_s_). The differences between ratios were calculated using a Wilcoxon signed rank test where the difference from a theoretical value (=1) was tested.

## Results

The patient material for this study was sampled on two separate occasions producing 20 samples to be used immediately after sampling (fresh) and then 28 samples of serum and platelet poor plasma to be frozen. Fourteen of the patients with frozen samples had been included in a previous study [19]. Clinical and demographical characteristics of the included SLE patients and healthy controls are presented in Table 1.

### ROS production and degranulation of PMNs stimulated with MPs and/or LPS

**Fig. 1:**
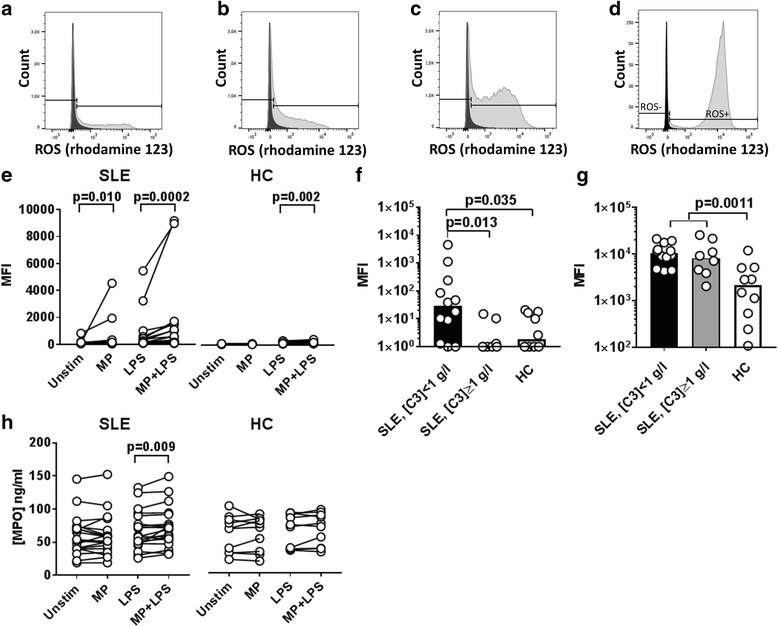
PMN ROS production and degranulation induced by autologous MPs and/or LPS. Leukocytes from 20 SLE patients and 10 healthy controls were incubated with (**a**) autologous MPs, (**b**) LPS, (**c**) a combination of autologous MPs and LPS or (**d**) PMA for 30 min in the presence of autologous serum and DHR, a probe for H_2_O_2_ production. Fluorescent oxidation product rhodamine 123 contained in PMNs staining positive for CD15 and negative for the dead cell marker near infrared was measured by flow cytometry and expressed as median fluorescence intensity (MFI) units. (**e**) Increase in ROS production after addition of MPs or MPs in combination with LPS. PMN production of ROS after stimulation with (**f**) MPs or (**g**) PMA, adjusted for background activity (of unstimulated cells), is shown for healthy controls and SLE patients with high and low levels of circulating complement C3, respectively. (**h**) Concentration of the primary granule protein myeloperoxidase (MPO) released into the cell supernatants. HC healthy control, LPS lipopolysaccharide, MP microparticle, ROS reactive oxygen species, SLE systemic lupus erythematosus, Unstim unstimulated

We examined ROS production by PMNs in fresh leukocyte preparations by flow cytometry using dihydrorhodamine 123 as a probe. The cells were stimulated with autologous MPs, alone or in combination with LPS, or with PMA (Fig. 1a–d). Within samples, the PMNs often showed heterogeneous ROS production after these stimuli. Incubation with MPs induced a significant increase in the production of ROS by PMNs from SLE patients but not by PMNs from healthy controls (Fig. 1e). When PMNs were stimulated with LPS, co-stimulation with MPs increased the production of ROS significantly in PMNs from both SLE patients and healthy controls (Fig. 1e).

We reasoned that the serum C3 level was a disease activity marker related to IC and MP clearance capacity in vivo. We therefore subdivided SLE patients into two groups with circulating C3 levels > 1 g/l and ≤ 1 g/l, respectively. Notably, ROS production by PMNs from patients with low circulating C3 levels exceeded that of both PMNs from patients with high C3 levels and that of healthy control PMNs after stimulation with MPs (Fig. 1f). An indication that this was due to hyper-responding PMNs in SLE patients came from the finding that PMNs from SLE patients produced significantly more ROS than PMNs from healthy controls after stimulation with PMA (*p* = 0.0011) (Fig. 1g). Using this stimulus, no difference was observed between high C3 and low C3 subgroups.

**Fig. 2:**
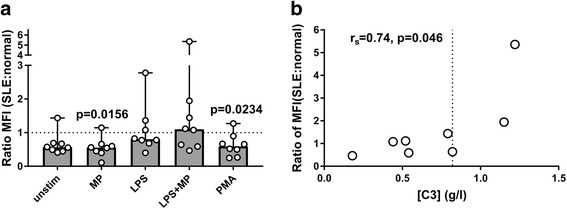
Influence of serum on MP-induced ROS production by SLE PMNs. Purified leukocytes from eight SLE patients, six of whom had SLEDAI > 5, were incubated with DHR and stimulated for 30 min at 37 °C with autologous MPs alone or in combination with LPS, or with PMA, in a medium containing 25% v/v autologous serum or a pool of NHS. The resulting ROS production by PMNs was analysed by flow cytometry, and the ratio between median fluorescence intensity (MFI) values obtained in the presence of SLE serum above that obtained in the presence of NHS was calculated after adjustment for background (unstimulated cells). Ratios from three experiments were excluded because either the numerator or denominator was negative. (**a**) Ratios between MFI values observed after stimulation with MPs and LPS in the presence of autologous serum above that observed in the presence of NHS. Bars represent median ratios. (**b**) Correlation between the ratio and circulating levels of C3 in samples stimulated with a combination of MPs and LPS. Dotted line represents the lower limit of the normal range. LPS lipopolysaccharide, MP microparticle, PMA phorbol-12-myristate-13-acetate, SLE systemic lupus erythematosus, Unstim unstimulated

We also examined degranulation, as another measure of PMN activation, and found that addition of autologous MPs to LPS-stimulated PMNs resulted in a significant increase in the release of MPO from primary granules in the SLE group, but not in the healthy control group (Fig. 2h). PMN degranulation of secondary or tertiary granules was not observed (see Additional file 2A, B).

### Influence of serum on ROS production and degranulation of PMNs

Having observed that PMNs from SLE patients showed higher responses to MPs than PMNs from healthy controls when autologous serum was present, we wished to examine to what extent this difference was determined by serum factors including autoantibodies and complement components. We therefore compared the production of ROS observed in autologous serum with that observed in NHS.

The ROS production induced by MPs and LPS was significantly higher in the presence of the SLE patients’ own serum than in the presence of NHS, as shown in Fig. 2a (*p* = 0.031). Conversely, the PMA-induced production of ROS by PMNs was decreased when SLE serum was present (*p* = 0.023).

An indication that consumption of complement in SLE serum influenced ROS production after stimulation of PMNs with MPs and LPS came from the finding that the SLE:NHS ratio for ROS production tended to correlate positively with the C3 content in the patients’ serum (*r*_s_ =0.75, *p* = 0.066) (Fig. 2b). By contrast, no correlation was observed with the serum content of antibodies to double-stranded deoxyribonucleic acid (dsDNA) (*r*_s_ = –0.22, *p* = 0.64) or with SLEDAI values (*r*_s_ = –0.31, *p* = 0.50). The origin of serum present during the stimulation with MP, alone or in combination with LPS, did not influence the PMN degranulation (see Additional file 2C–E).

**Fig. 3:**
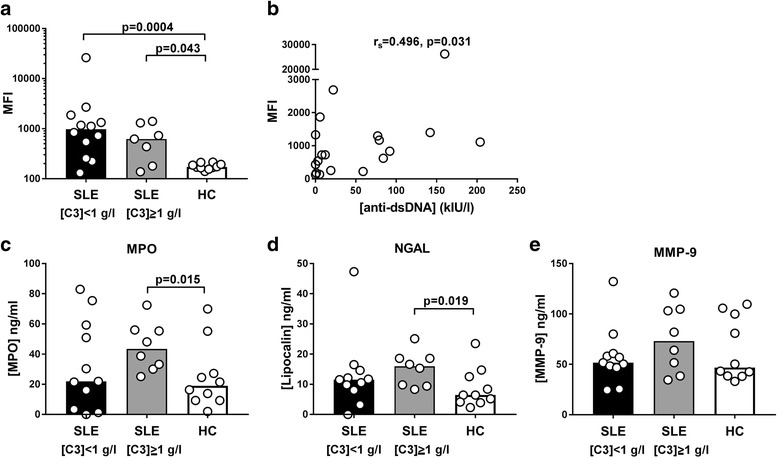
MP-induced ROS production and degranulation of PMNs in the presence of heterologous sera. Leukocytes from a healthy blood group 0 donor were suspended in a medium containing 25% v/v of serum from 20 SLE patients and 10 healthy controls (see Table 1, frozen samples). DHR was used as probe for H_2_O_2_, and the cells were stimulated with autologous MPs in combination with LPS for 30 min at 37 °C before flow cytometry. (**a**) Median fluorescence intensity (MFI) after subtraction of background fluorescence (unstimulated cells). SLE patients were divided into two groups with circulating C3 levels > 1 g/l and ≤ 1 g/l, respectively. (**b**) Correlation between MFI values and anti-dsDNA antibody levels of patient sera. (**c**–**e**) Degranulation measured in the corresponding cell supernatants as the content of myeloperoxidase (MPO) from primary granules, neutrophil gelatinase-associated lipocalin (NGAL) from secondary granules and matrix metallopeptidase 9 (MMP-9) from tertiary granules. Bars represent median values. dsDNA double-stranded DNA, HC healthy control, SLE systemic lupus erythematosus

To test directly the effect of serum on MP-induced ROS production by PMNs, leukocytes from a single healthy control were stimulated with a combination of autologous MPs and LPS in the presence of heterologous sera derived from 20 patients (10 of whom had active disease) and 10 healthy controls (Fig. 3). Under these circumstances, PMN production of ROS was greatly promoted by sera from SLE patients, irrespective of the C3 content, compared with sera from healthy controls (*p* = 0.0034) (Fig. 3a). The ROS production correlated positively with the level of circulating anti-dsDNA antibodies in SLE serum (*r*_s_ = 0.496, *p* = 0.0031) (Fig. 3b) but not with the level of circulating C3 (see Additional file 3). Release of both primary and secondary granules increased when SLE serum with high C3 content was present (Fig. 3c, d). A similar tendency was observed for the release of tertiary granules (Fig. 3e).

### Influence of antibodies on MP-induced ROS production by PMNs

**Fig. 4:**
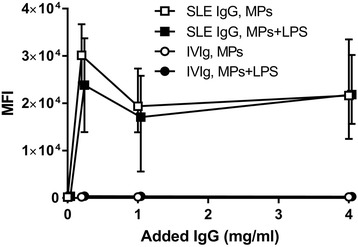
Influence of exogenously added IgG on MP-induced ROS production by PMNs. Leukocytes purified from three healthy blood group 0 donors were stimulated with autologous MPs, alone (open symbols) or in combination with LPS (closed symbols), in the presence of autologous serum supplemented with various amounts of IgG from SLE patients (SLE IgG) or healthy donors (IVIg). DHR was used as probe for H_2_O_2_ production. Shown is the resulting median fluorescence intensity (MFI) of PMNs, as measured by flow cytometry. Symbols represent average and error bars represent range. IgG immunoglobulin G, LPS lipopolysaccharide, MP microparticle, SLE systemic lupus erythematosus

To investigate the influence of antibodies in the serum-mediated enhancement of ROS production, leukocytes from healthy controls were stimulated with autologous MPs, alone or in combination with LPS, in the presence of autologous serum supplemented with various amounts of either IgG purified from SLE patients or IVIg purified from healthy donors. As shown in Fig. 4, IgG from SLE patients strongly promoted ROS production by PMNs.

### Influence of MPs on ROS production and degranulation by normal PMNs

**Fig. 5:**
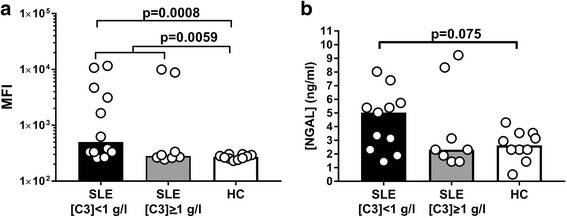
Influence of SLE and healthy control MPs on MP-induced ROS production and degranulation of PMNs. Leukocytes from a healthy control were suspended in a medium containing 25% v/v of normal human serum. DHR was added to the suspensions, and the cells were stimulated with LPS in combination with MPs from 20 SLE patients and 10 healthy controls for 30 min at 37 °C before flow cytometry. (**a**) The resulting median fluorescence intensity (MFI) after subtraction of background fluorescence (unstimulated cells). Bars represent median values. Comparisons between groups using Mann–Whitney *U* test (**b**) Content of neutrophil gelatinase-associated lipocalin (NGAL) in the supernatants of leukocytes stimulated with MPs and LPS in combination shown as median values after subtraction of the background (unstimulated cells). HC healthy control, SLE systemic lupus erythematosus

To examine whether MPs from SLE patients and healthy controls differed in their capacity to induce production of ROS in PMNs, we compared the ROS production by normal PMNs stimulated with LPS and MPs derived from 20 SLE patients and 10 healthy controls. MPs from SLE patients induced more ROS production than did MPs from healthy controls, and this was statistically significant for the total group of SLE patients (*p* = 0.006) and for those with high disease activity, as measured by the level of circulating C3 (*p* = 0.0008), but not for those with a correspondingly low disease activity (Fig. 5a). MPs from SLE patients and healthy controls did not differ in their ability to induce release of primary or tertiary granule contents by PMNs when co-incubated with LPS (see Additional file 2F, G). However, MPs from SLE patients with complement activation tended to induce release of more NGAL from secondary granules than MPs from healthy controls (Fig. 5b).

## Discussion

The objective of this study was to examine the ability of plasma-derived MPs to activate PMNs for production of ROS and degranulation, and to compare these effects in SLE patients and healthy controls.

A key finding was that MPs from SLE patients induced ROS production in the patients’ own PMNs suspended in autologous serum, thus resembling in-vivo conditions. The same was not observed when PMNs from healthy controls suspended in autologous serum were stimulated with MPs purified from their own blood. This supports the notion that MPs mediate pro-inflammatory effects in SLE. The MP-induced ROS production was the most pronounced in patients with low levels of circulating C3. As low C3 levels may reflect complement activation in vivo, these are generally considered markers of active disease in SLE patients [27, 28].

At least three factors may have contributed to the increased responsiveness of SLE PMNs to MPs in the autologous setting: the PMNs of SLE patients, particularly those with low C3 levels, may have been primed in vivo; serum components (e.g. opsonizing autoantibodies or increased levels of anaphylatoxins or pro-inflammatory cytokines) may promote PMN activation; and the MPs per se may have increased capacity for PMN activation (e.g. due to tagging with complement fragments [19] or antibodies [23]).

An indication that patient PMNs had been primed in vivo came from our finding that PMNs from SLE patients showed increased responsiveness to PMA. In accordance, Dieker et al. [22] demonstrated increased NETosis by SLE PMNs after stimulation with in-vivo-generated MPs and LPS. Our findings are in contrast with previous findings of decreased ROS production after stimulation of PMNs from SLE patients with PMA [8] or with complement-opsonized ICs [9]. The former study used PMNs stored for up to 24 hours prior to the experiment, which might have caused death of the most pre-activated PMNs. SLE is associated with increased PMN death via both apoptosis and NETosis [7]. Increased propensity of PMNs from SLE patients to produce ROS may, at least in part, be explained by decreased expression of FcγRII, which inhibits ROS production [29, 30].

The influence of serum factors on MP-induced PMN responses was assessed in three ways in this study. Firstly, the response of SLE PMNs to autologous MPs in the presence of autologous serum was compared with that observed in the presence of NHS. Secondly, PMNs from a single healthy donor were stimulated with autologous MPs in combination with LPS, in a medium containing serum from 20 different SLE patients and 10 healthy controls. Both approaches revealed that SLE sera promoted ROS production by PMNs, and the latter revealed an association between MP-induced ROS production and circulating anti-dsDNA levels. Thirdly, a role for antibodies in the serum-mediated enhancement of ROS production by PMNs was demonstrated by the finding that IgG from SLE serum markedly increased the ROS production induced by MPs alone or in combination with LPS. Previous studies have shown that SLE serum is able to induce ROS production by healthy PMNs without any additional stimuli, and because this also applied to complement-depleted serum, autoantibodies were assumed to be the main stimulant [31, 32].

We also examined the influence of MPs per se on PMN ROS production. The combination of LPS and MPs from patients with low circulating C3 levels induced more ROS than LPS in combination with MPs from healthy controls or patients with relatively high C3 levels. Low levels of circulating C3 presumably reflect complement consumption as a result of complement activation in vivo. We have not measured C3 and IgG on MPs derived from the NHDs and SLE patients included in this study, but we have in our unit previously examined the composition of MPs from healthy donors and SLE patients using the same MP purification technique: We found that MPs from SLE patients had increased amounts of C3 fragments and IgG on the surface [19, 23], and increased deposition of complement on MPs has been associated with disease activity [33]. Moreover, increased amounts of IgG on the MP surface are related to disease activity [23]. This IgG deposition may, via interaction with FcRs in the same manner as ICs [29], further enhance the ability of SLE MPs to induce ROS in PMNs. We therefore find it very likely that in-vivo tagging of MPs from SLE patients with complement and IgG is responsible for the increased ability of these MPs to induce production of ROS by PMNs.

The PMN degranulation did not differ between SLE patients and healthy controls in the autologous setting. Stimulation with MPs in combination with LPS induced release of primary granules, as measured by MPO release, in both groups, but only in the SLE group did MPs enhance LPS-stimulated MPO release. An indication that SLE serum may contain factors which promote release of both primary and secondary granules came from the finding that the combination of MPs and LPS seemed to induce release of more MPO and NGAL in the presence of SLE patient serum containing normal C3 levels than in the presence of SLE serum containing low C3 levels. It is likely that the factor in SLE serum promoting degranulation is autoantibodies. Activation of FcγRIII plays an important role in degranulation [29]. However, the fact that degranulation was related to the presence of C3 also corresponds well with the co-stimulatory role demonstrated for CR3 in induction of degranulation [29].

Limitations of this study are the low numbers of highly active SLE patients (including those with nephritis) and the lack of a disease control group. The number of included patients was limited. Nevertheless, we were able to demonstrate clear differences between SLE patients and healthy controls, and between patients with high and low circulating C3 levels. Further research should include more mechanistic studies, such as investigation of the role of complement receptors and ICs bound to the MP surface in MP-induced stimulation of PMNs.

## Conclusions

The present study demonstrates that PMNs from SLE patients are hyper-responsive to stimulation with MPs or PMA, and that MPs, serum and purified IgG from SLE patients each promote ROS production by PMNs. These factors may all contribute to driving ROS-dependent inflammation in SLE.

## Additional files

Additional file 1: Is a figure showing flow cytometric gating of live PMNs. PMNs in samples of leukocytes were identified on the basis of **(A)** forward and side scatter characteristics, **(B)** gating for single cells and **(C)** positive staining for CD15 and negative staining for the dead cell marker near infrared (IR). (JPG 203 kb)

Additional file 2: Is a figure showing degranulation of PMNs stimulated with MPs in the presence of serum. **(A, B)** Leukocytes from 20 SLE patients and 10 healthy controls were incubated with autologous MPs, LPS or a combination of autologous MPs and LPS for 30 min in the presence of autologous serum. Concentration of **(A)** neutrophil gelatinase-associated lipocalin (NGAL) from secondary granules and **(B)** matrix metallopeptidase 9 (MMP-9) from tertiary granules in cell supernatants. **(C–E)** Leukocytes from eight SLE patients were incubated for 30 min at 37 °C with autologous MPs alone or in combination with LPS, or with PMA, in a medium containing 25% v/v autologous serum or normal human serum (NHS). Concentrations of **(C)** myeloperoxidase (MPO) from primary granules, **(D)** NGAL from secondary granules and **(E)** MMP-9 from tertiary granules in the presence of SLE serum above that observed when NHS was present (SLE:normal) and after subtraction of background (unstimulated cells). **(F, G)** Leukocytes from a healthy control were incubated with LPS in combination with MPs from 20 SLE patients and 10 healthy controls in the presence of NHS for 30 min at 37 °C. Contents of **(F)** MPO and **(G)** MMP-9 in the supernatants shown as median values after subtraction of background (unstimulated cells). Bars represent median values. Granule contents measured by Luminex assays. (JPG 1139 kb)

Additional file 3: Is a figure showing MP-induced ROS production by PMNs in the presence of SLE sera. Leukocytes from a healthy blood group 0 donor were suspended in a medium containing 25% v/v of serum from 20 SLE patients (see Table 1, frozen samples). DHR was used as probe for H_2_O_2_, and the cells were stimulated with autologous MPs in combination with LPS for 30 min at 37 °C before flow cytometry. Correlation between the resulting median fluorescence intensity (MFI) after subtraction of background fluorescence (unstimulated cells) and levels of circulating anti-dsDNA antibodies. (JPG 175 kb)

## Abbreviations

dsDNA: Double-stranded deoxyribonucleic acid
FcγR: Fc gamma receptor
IgG: Immunoglobulin G
LPS: Lipopolysaccharide
MP: Microparticle
NETosis: Production of neutrophil extracellular traps
PMA: Phorbol-12-myristate-13-acetate
PMN: Polymorphonuclear leukocyte
ROS: Reactive oxygen species
SLE: Systemic lupus erythematosus

## References

[1] Sisirak V, Sally B, Agati VD, Clancy RM, Buyon JP, Reizis B. Digestion of chromatin in apoptotic cell microparticles prevents autoimmunity. Cell. 2016;166:88–101. 10.1016/j.cell.2016.05.034 PMC5030815 27293190

[2] Beyer C, Pisetsky DS (2010). The role of microparticles in the pathogenesis of rheumatic diseases. Nat Rev Rheumatol..

[3] Pereira J, Alfaro G, Goycoolea M, Quiroga T, Ocqueteau M, Massardo L (2006). Circulating platelet-derived microparticles in systemic lupus erythematosus. Association with increased thrombin generation and procoagulant state. Thromb Haemost.

[4] Nielsen CT, Østergaard O, Johnsen C, Jacobsen S, Heegaard NHH (2011). Distinct features of circulating microparticles and their relationship to clinical manifestations in systemic lupus erythematosus. Arthritis Rheum..

[5] Ullal AJ, Reich CF, Clowse M, Criscione-Schreiber LG, Tochacek M, Monestier M (2011). Microparticles as antigenic targets of antibodies to DNA and nucleosomes in systemic lupus erythematosus. J Autoimmun..

[6] Nielsen CT, Østergaard O, Rekvig OP, Sturfelt G, Jacobsen S, Heegaard NHH (2015). Galectin-3 binding protein links circulating microparticles with electron dense glomerular deposits in lupus nephritis. Lupus..

[7] Kaplan MJ (2011). Neutrophils in the pathogenesis and manifestations of SLE. Nat Rev Rheumatol..

[8] Bengtsson AA, Pettersson Å, Wichert S, Gullstrand B, Hansson M, Hellmark T (2014). Low production of reactive oxygen species in granulocytes is associated with organ damage in systemic lupus erythematosus. Arthritis Res Ther..

[9] Nielsen CH, Rasmussen JM, Voss A, Junker P, Leslie RG (1998). Diminished ability of erythrocytes from patients with systemic lupus erythematosus to limit opsonized immune complex deposition on leukocytes and activation of granulocytes. Arthritis Rheum..

[10] Perazzio SF, Salomão R, Silva NP, Andrade LEC (2012). Increased neutrophil oxidative burst metabolism in systemic lupus erythematosus. Lupus..

[11] Molad Y, Buyon J, Anderson DC, Abramson SB, Cronstein BN (1994). Intravascular neutrophil activation in systemic lupus erythematosus (SLE): dissociation between increased expression of CD11b/CD18 and diminished expression of L-selectin on neutrophils from patients with active SLE. Clin Immunol Immunopathol..

[12] Perl A (2013). Oxidative stress in the pathology and treatment of systemic lupus erythematosus. Nat Rev Rheumatol..

[13] Bergamo P, Maurano F, Rossi M (2007). Phase 2 enzyme induction by conjugated linoleic acid improves lupus-associated oxidative stress. Free Radic Biol Med..

[14] Lai Z-W, Hanczko R, Bonilla E, Caza TN, Clair B, Bartos A (2012). N-acetylcysteine reduces disease activity by blocking mammalian target of rapamycin in T cells from systemic lupus erythematosus patients: a randomized, double-blind, placebo-controlled trial. Arthritis Rheum..

[15] Nielsen CH, Antonsen S, Matthiesen SH, Leslie RG (1997). The roles of complement receptors type 1 (CR1, CD35) and type 3 (CR3, CD11b/CD18) in the regulation of the immune complex-elicited respiratory burst of polymorphonuclear leukocytes in whole blood. Eur J Immunol..

[16] Zhou MJ, Brown EJ (1994). CR3 (Mac-1, alpha M beta 2, CD11b/CD18) and Fc gamma RIII cooperate in generation of a neutrophil respiratory burst: requirement for Fc gamma RIII and tyrosine phosphorylation. J Cell Biol..

[17] Clemmensen SN, Udby L, Borregaard N (2014). Subcellular fractionation of human neutrophils and analysis of subcellular markers. Methods Mol Biol..

[18] Huizinga TW, Dolman KM, van der Linden NJ, Kleijer M, Nuijens JH, von dem Borne AE (1990). Phosphatidylinositol-linked FcRIII mediates exocytosis of neutrophil granule proteins, but does not mediate initiation of the respiratory burst. J Immunol..

[19] Winberg LK, Nielsen CH, Jacobsen S (2017). Surface complement C3 fragments and cellular binding of microparticles in patients with SLE. Lupus Sci Med..

[20] Jy W, Mao WW, Horstman L, Tao J, Ahn YS. Platelet microparticles bind, activate and aggregate neutrophils in vitro. Blood Cells Mol Dis. 1995;21:217–31. discussion 231a. 10.1006/bcmd.1995.0025 8673474

[21] Sadallah S, Eken C, Martin PJ, Schifferli JA (2011). Microparticles (ectosomes) shed by stored human platelets downregulate macrophages and modify the development of dendritic cells. J Immunol..

[22] Dieker J, Tel J, Pieterse E, Thielen A, Rother N, Bakker M (2016). Circulating apoptotic microparticles in systemic lupus erythematosus patients drive the activation of dendritic cell subsets and prime neutrophils for NETosis. Arthritis Rheumatol..

[23] Nielsen CT, Østergaard O, Stener L, Iversen LV, Truedsson L, Gullstrand B (2012). Increased IgG on cell-derived plasma microparticles in systemic lupus erythematosus is associated with autoantibodies and complement activation. Arthritis Rheum..

[24] Tan EM, Cohen AS, Fries JF, Masi AT, Mcshane DJ, Rothfield NF (1982). The 1982 revised criteria for the classification of systemic lupus erythematosus. Arthritis Rheum..

[25] Hochberg MC (1997). Updating the American College of Rheumatology revised criteria for the classification of systemic lupus erythematosus. Arthritis Rheum..

[26] Touma Z, Urowitz MB, Ibañez D, Gladman DD (2011). SLEDAI-2 K 10 days versus SLEDAI-2 K 30 days in a longitudinal evaluation. Lupus..

[27] Arriens C, Wren JD, Munroe ME, Mohan C. Systemic lupus erythematosus biomarkers: the challenging quest. Rheumatology. 2017;56:i32–i45. 10.1093/rheumatology/kew407 PMC5850341 28013203

[28] Pickering MC, Walport MJ (2000). Links between complement abnormalities and systemic lupus erythematosus. Rheumatology (Oxford).

[29] Marzocchi-Machado CM, Alves CMOS, Azzolini a ECS, Polizello a CM, Carvalho IF, Lucisano-Valim YM. Fcgamma and complement receptors: expression, role and co-operation in mediating the oxidative burst and degranulation of neutrophils of Brazilian systemic lupus erythematosus patients. Lupus. 2002;11:240–8 10.1191/0961203302lu172oa 12043888

[30] van Lent P, Nabbe KC, Boross P, Blom AB, Roth J, Holthuysen A (2003). The inhibitory receptor FcgammaRII reduces joint inflammation and destruction in experimental immune complex-mediated arthritides not only by inhibition of FcgammaRI/III but also by efficient clearance and endocytosis of immune complexes. Am J Pathol..

[31] Shingu M, Oribe M, Todoroki T, Tatsukawa K, Tomo-Oka K, Yasuda M (1983). Serum factors from patients with systemic lupus erythematosus enhancing superoxide generation by normal neutrophils. J Invest Dermatol..

[32] Hashimoto Y, Ziff M, Hurd ER (1982). Increased endothelial cell adherence, aggregation, and superoxide generation by neutrophils incubated in systemic lupus erythematosus and felty’s syndrome sera. Arthritis Rheum..

[33] Østergaard O, Nielsen CT, Iversen LV, Tanassi JT, Knudsen S, Jacobsen S (2013). Unique protein signature of circulating microparticles in systemic lupus erythematosus. Arthritis Rheum..

