# Supplementary figures and images for: Microparticles from patients with systemic lupus erythematosus induce production of reactive oxygen species and degranulation of polymorphonuclear leukocytes
Source: Arthritis Res Ther. 2017 Oct 17;19:230. doi: 10.1186/s13075-017-1437-3 (PMC5646131; doi:10.1186/s13075-017-1437-3)

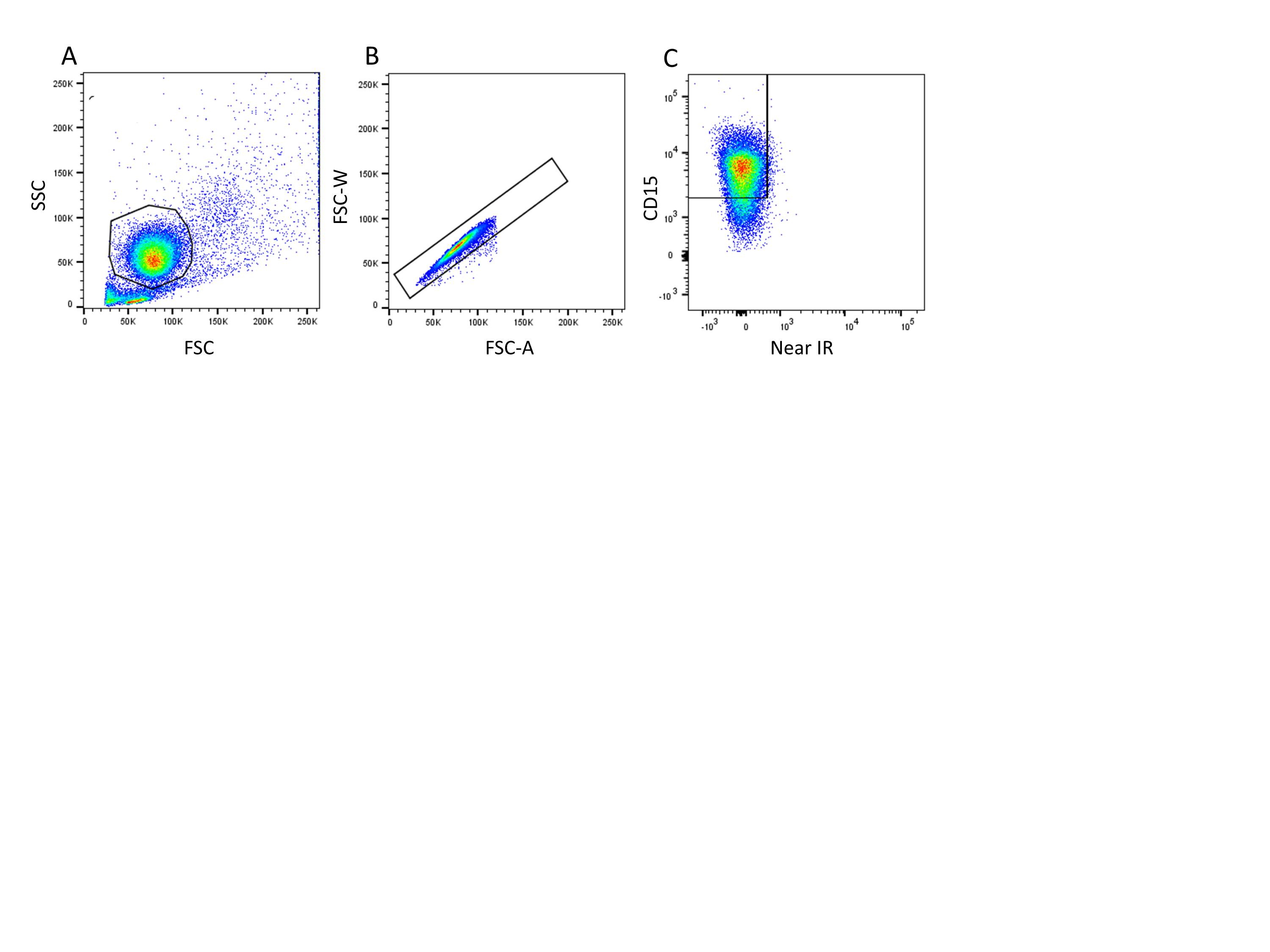

Supplement: Supplementary file 1 — Is a figure showing flow cytometric gating of live PMNs. PMNs in samples of leukocytes were identified on the basis of (A) forward and side scatter characteristics, (B) gating for single cells and (C) positive staining for CD15 and negative staining for the dead cell marker near infrared (IR). (JPG 203 kb) [file 13075_2017_1437_MOESM1_ESM.jpg]

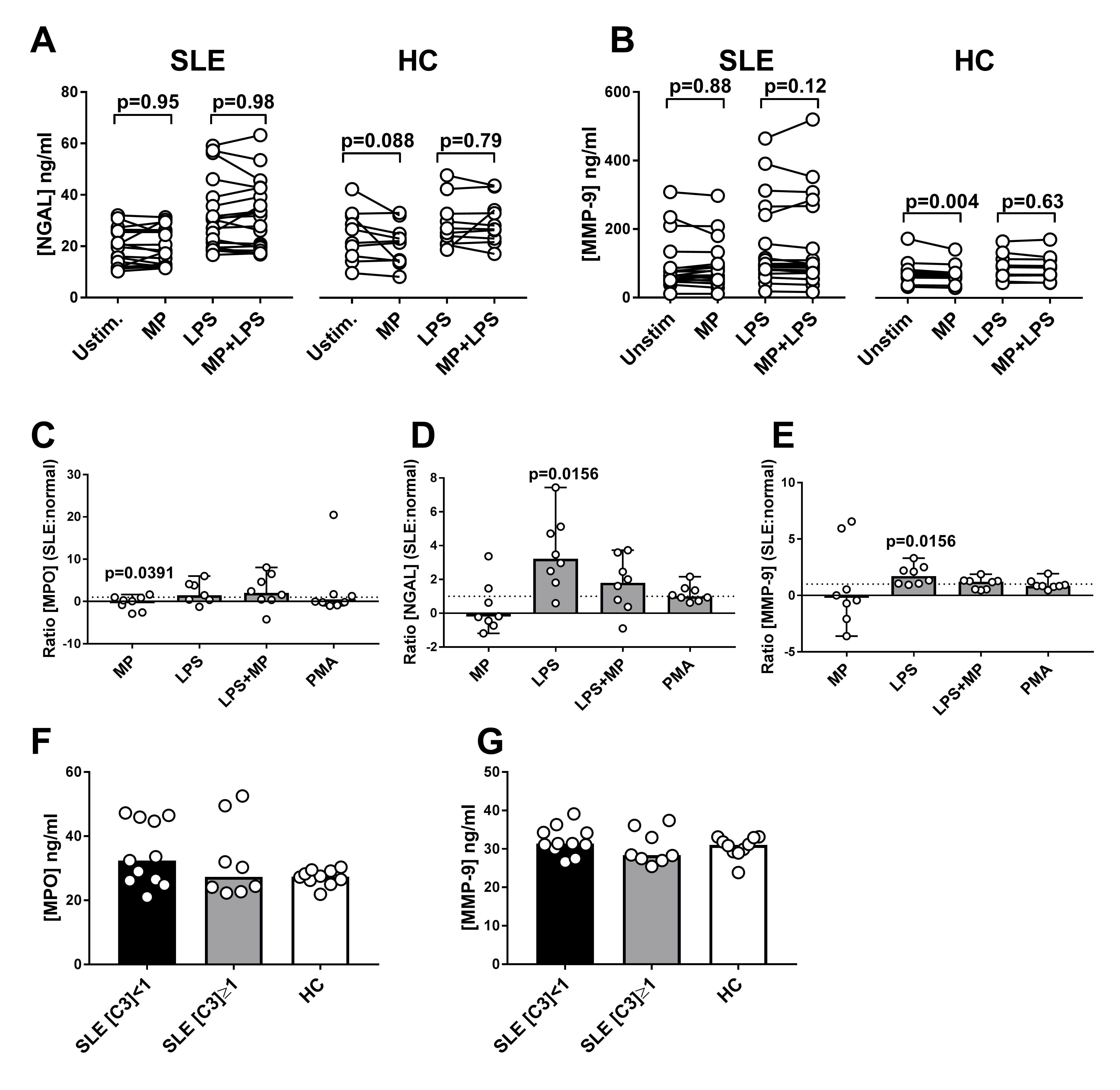

Supplement: Supplementary file 2 — Is a figure showing degranulation of PMNs stimulated with MPs in the presence of serum. (A, B) Leukocytes from 20 SLE patients and 10 healthy controls were incubated with autologous MPs, LPS or a combination of autologous MPs and LPS for 30 min in the presence of autologous serum. Concentration of (A) neutrophil gelatinase-associated lipocalin (NGAL) from secondary granules and (B) matrix metallopeptidase 9 (MMP-9) from tertiary granules in cell supernatants. (C–E) Leukocytes from eight SLE patients were incubated for 30 min at 37 °C with autologous MPs alone or in combination with LPS, or with PMA, in a medium containing 25% v/v autologous serum or normal human serum (NHS). Concentrations of (C) myeloperoxidase (MPO) from primary granules, (D) NGAL from secondary granules and (E) MMP-9 from tertiary granules in the presence of SLE serum above that observed when NHS was present (SLE:normal) and after subtraction of background (unstimulated cells). (F, G) Leukocytes from a healthy control were incubated with LPS in combination with MPs from 20 SLE patients and 10 healthy controls in the presence of NHS for 30 min at 37 °C. Contents of (F) MPO and (G) MMP-9 in the supernatants shown as median values after subtraction of background (unstimulated cells). Bars represent median values. Granule contents measured by Luminex assays. (JPG 1139 kb) [file 13075_2017_1437_MOESM2_ESM.jpg]

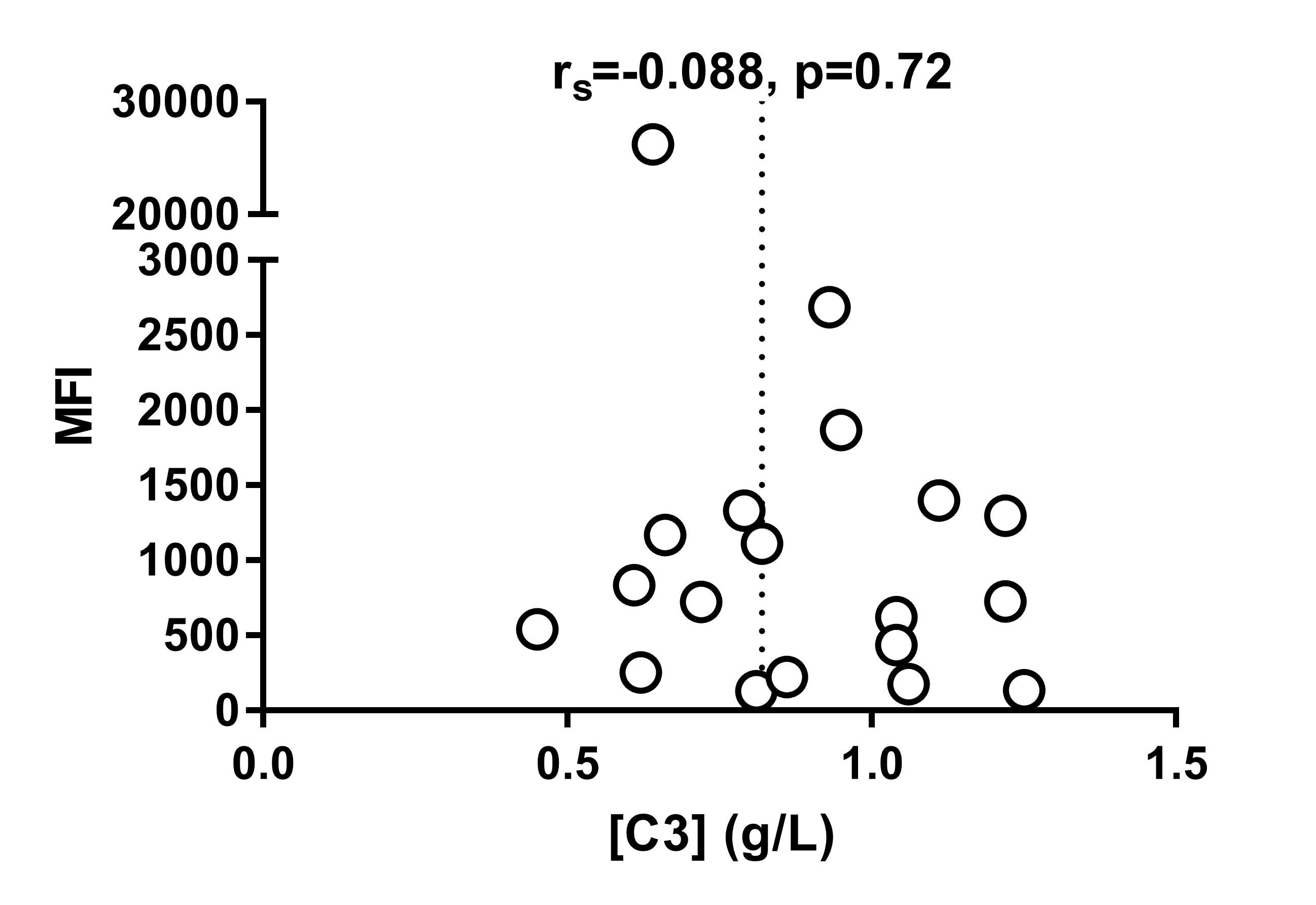

Supplement: Supplementary file 3 — Is a figure showing MP-induced ROS production by PMNs in the presence of SLE sera. Leukocytes from a healthy blood group 0 donor were suspended in a medium containing 25% v/v of serum from 20 SLE patients (see Table 1, frozen samples). DHR was used as probe for H2O2, and the cells were stimulated with autologous MPs in combination with LPS for 30 min at 37 °C before flow cytometry. Correlation between the resulting median fluorescence intensity (MFI) after subtraction of background fluorescence (unstimulated cells) and levels of circulating anti-dsDNA antibodies. (JPG 175 kb) [file 13075_2017_1437_MOESM3_ESM.jpg]
